# Ubiquitinated proteins enriched from tumor cells by a ubiquitin binding protein Vx3(A7) as a potent cancer vaccine

**DOI:** 10.1186/s13046-015-0156-3

**Published:** 2015-04-16

**Authors:** Mohanad Aldarouish, Huzhan Wang, Meng Zhou, Hong-Ming Hu, Li-xin Wang

**Affiliations:** Department of Microbiology and Immunology, Medical School of Southeast University, 87 Dingjiaqiao Rd, Nanjing, Jiangsu Province 210009 People’s Republic of China; Laboratory of Cancer Immunobiology, Earle A. Chiles Research Institute, Providence Portland Medical Center, Portland, Oregon USA

**Keywords:** Vx3(A7) protein, Ubiquitinated proteins, Tumor-derived autophagosomes (DRibbles), Antigen cross-presentation, Anti-tumor efficacy

## Abstract

**Background:**

Our previous studies have demonstrated that autophagosome-enriched vaccine (named DRibbles: DRiPs-containing blebs) induce a potent anti-tumor efficacy in different murine tumor models, in which DRibble-containing ubiquitinated proteins are efficient tumor-specific antigen source for the cross-presentation after being loaded onto dendritic cells. In this study, we sought to detect whether ubiquitinated proteins enriched from tumor cells could be used directly as a novel cancer vaccine.

**Methods:**

The ubiquitin binding protein Vx3(A7) was used to isolate ubiquitinated proteins from EL4 and B16-F10 tumor cells after blocking their proteasomal degradation pathway. C57BL/6 mice were vaccinated with different doses of Ub-enriched proteins via inguinal lymph nodes or subcutaneous injection and with DRibbles, Ub-depleted proteins and whole cell lysate as comparison groups, respectively. The lymphocytes from the vaccinated mice were re-stimulated with inactivated tumor cells and the levels of IFN-γ in the supernatant were detected by ELISA. Anti-tumor efficacy of Ub-enriched proteins vaccine was evaluated by monitoring tumor growth in established tumor mice models. Graphpad Prism 5.0 was used for all statistical analysis.

**Results:**

We found that after stimulation with inactivated tumor cells, the lymphocytes from the Ub-enriched proteins-vaccinated mice secreted high level of IFN-γ in dose dependent manner, in which the priming vaccination via inguinal lymph nodes injection induced higher IFN-γ level than that via subcutaneous injection. Moreover, the level of secreted IFN-γ in the Ub-enriched proteins group was markedly higher than that in the whole cell lysate and Ub-depleted proteins. Interestingly, the lymphocytes from mice vaccinated with Ub-enriched proteins, but not Ub-depleted proteins and whole cell lysates, isolated from EL4 or B16-F10 tumor cells also produced an obvious level of IFN-γ when stimulated alternately with inactivated B16-F10 or EL4 tumor cells. Furthermore, Ub-enriched proteins vaccine showed a significant inhibitory effect on in vivo growth of homologous tumor, as well as allogeneic tumor, compared with Ub-depleted proteins and tumor cell lysate. Tumor growth was regressed after three times of vaccination with Ub-enriched proteins in contrast to other groups.

**Conclusion:**

These results indicated that Ub-enriched proteins isolated from tumor cells may have a potential as a potent vaccine for immunotherapy against cancer.

## Background

Cross-presentation is the process by which the antigens from “antigen-donor cell (ADC)” are captured, processed, and then presented to antigen-specific T cells by host professional antigen-presenting cells (pAPCs) [1-3]. It is well established that cross-presentation of tumor antigens derived from tumor cell as antigen-donor cell plays a pivotal role in the initiation and development of cytotoxic T lymphocytes (CD8^+^ CTL) immune response to tumor-associated antigens (TAAs), including self or mutated self-antigens derived from tumor cells [1,4].

There are two major pathways for TAAs proteolysis in the tumor cells. The short-lived proteins (SLiPs), including the defective ribosomal products (DRiPs), are ubiquitinated and degraded by proteasomes [5,6], whereas the long-lived proteins are mostly degraded by the lysosomes through the autophagy pathway [7,8]. It is generally believed that the proteasome-mediated protein degradation pathway plays an important role in providing peptides for MHC-I restricted antigen presentation in antigen-donor cells (direct presentation), while the long-lived protein but not short-lived proteins are normally cross-presented by host pAPCs. Under abnormal physiological conditions, i.e., when either pathway is defective, the degradation of proteins is shunted from one pathway to the other to protect cell survival [9].

Our previous studies have demonstrated that with induction of autophagy and inhibition of lysosomal/proteosomal activity, a broad spectrum of cellular antigens, including long-lived proteins, short-lived proteins, as well as defective ribosomal products, are sequestered in autophagosomes [9]. We refer these autophagosome-enriched, defective ribosomal products-containing blebs as to DRibbles. We also documented that DRibbles derived from tumor cells are efficient TAAs carriers for cross-presentation by dendretic cells, by which DRibble vaccine can stimulate dramatic T-cell activation, leading to an anti-tumor efficacy in different tumor models such as melanomas, lung carcinomas, breast carcinomas and liver cancer [10-12].

It’s believed that DRibble can serve as a vessel to ferry a broad spectrum of tumor antigens to pAPCs for efficient cross-presentation, and the anti-tumor efficacy induced by DRibbles is mainly depending on its content of ubiquitinated TAAs [9]. In this study, we sought to isolate ubiquitinated proteins (Ub-Ps), including short-lived proteins, as well as defective ribosomal products, from tumor cells after inhibition of their proteasome-mediated protein degradation pathway, and detect whether Ub-Ps could be used directly as a novel cancer vaccine to induce a specific tumor immune response.

## Materials and methods

### Cell culture and reagents

The following two cancer cell lines, lymphoma EL4 and melanoma B16-F10 were cultured in PRMI 1640 and DMEM medium respectively supplemented with 10% heat-inactivated fetal bovine serum, 100 U/ml penicillin, 0.1 mg/ml streptomycin (Beyotime Institute of Biotechnology, Haimen, China) in a humidified incubator in 5% CO2 at 37°C. pUbiG101-Vx3(A7)-eGFP plasmid was kindly gifted by Prof. Hong-Ming Hu (Providence Cancer Center, Providence Portland Medical Center, USA). Ni-NTA agarose beads were purchased from MCLAB Company (NINTA-200).

### Mice

C57BL/6 female mice were purchased from the Comparative Medicine Center, Yangzhou University (Yangzhou, China). All mice were bred and maintained in a specific pathogen-free condition. All experimental protocols were approved by the Institutional Animal Care and Use Committee of Southeast University.

### Tumor cell lysate preparation

Proteasome degradation pathway in tumor cells was blocked by adding 100 nM Bortezomib (Velcade) in complete medium for 5 hours in a 5% CO2 incubator at 37°C. The treated cells were centrifuged (1200 rpm/4°C-SORVALL ST 16R) for 10 minutes, and washed three times by PBS. The precipitated cells were then lysed with a Tris-base lysis buffer containing 20 mM Tris-base, 137 mM NaCl, 1% NP-40, 20 mM EDTA and protease/phosphatase inhibitor cocktail for 30 minutes. Cell lysates were collected after centrifuge (14000 rpm/4°C-Eppendorf AG 5417R) for 15 minutes [13]. Protein concentration was quantified by BCA protein assay Kit according to the manufacturer’s protocol (Beyotime Institute of Biotechnology).

### Vx3(A7) protein expression and purification

pUbiG101-Vx3(A7)-eGFP expressing vector was cloned into E. coli DH5-α competent cells (invitrogen). Cells were grown in LB medium for 3 hours at 37°C (until OD550 = 0.5**)**. After that, 1 M IPTG was added for 16 hrs at 15°C. Cells were harvested and centrifuged (8000 rpm/4°C-himac CR 21G) for 15 minutes. Pellet was re-suspended in sodium-phosphate buffer supplied with 0.001 mg/ml lysozyme and kept in ice for 30 minutes. After sonication of the solution using six 10-seconds bursts at high intensity, cellular debris in the lysate were pelleted down through centrifugation (15000 rpm/4°C-himac CR 21G) for 10 minutes. Supernatant was collected as ‘Bacterial cell lysate’ sample.

Vx3(A7) protein was purified using Ni-NTA agarose beads. Briefly, beads were washed three times by native binding buffer after washing with sterile distilled water. The beads were incubated with the previous Bacterial cell lysate for 1 hour at 4°C with gentle agitation. Unbound proteins were collected as ‘Flowing fraction’ followed by washing beads with the native washing buffer for 3 times and collected as ‘Washing fraction’. The bounded proteins were eluted by native elution buffer (0.25 M Imidazole) as ‘Elution fraction’. The purity of eluted proteins was tested by SDS PAGE. The endotoxin level of proteins used for vaccination was < 0.2 EU/mg.

### Ubiquitinated proteins purification

Ni-NTA agarose beads were washed one time with sterile distilled water followed by three washing times with the native binding buffer and then were incubated with the purified Vx3(A7) protein for 1 hour at 4°C. Agarose beads bounded with Vx3(A7) protein were washed three times by native washing buffer and then were incubated with the prepared whole tumor cell lysate for 24 hours at 4°C. Unbound fractions were collected and beads were washed three times by native washing buffer. Vx3(A7)-Ubiquitinated proteins complex was eluted using native elution buffer (3 M Imidazole). Eluted proteins were tested by western blot.

### Western blotting

The samples were resolved by 12.5% SDS-PAGE (Invitrogen) after mixing with SDS-PAGE loading buffer and boiling for 5 minutes. Proteins were transferred to a nitrocellulose membrane and diluted in blocking buffer (5% dry milk) for 1 hour, and then incubated separately with mouse anti-ubiquitin antibody (1:500, Sigma) and anti-K63 antibody (1:500, Enzo) overnight. Secondary antibody (1:5000, Pierce) was added for 1 hour. Membrane was exposed using the cassette in dark room.

### DRibbles preparation

Tumor cells were treated with 100 nM Rapamycin (Enzo Life Sciences, BML-A275-0005), 100 nM Bortezomib, and 10 mM ammonium chloride in complete medium for 18–24 hours in a 5% CO2 incubator at 37°C. The cells and the supernatant were harvested and spun at (1300 rpm-SORVALL ST 16R). The supernatant was then spun at (30,000 rpm/4°C-himac CR 21G) to harvest the DRibbles. The total concentration of DRibbles proteins was measured by Bradford assay [14].

### Detection of immune responses and immunotherapy experiments

C57BL/6 female mice were vaccinated three times with two days of intervals via two different routes of administration, subcutaneous injection (s.c.) or inguinal lymph nodes injection (i.n.) and with different doses (0, 30, 100 and 300 μg total protein / mouse) of whole cell lysate, Ub-depleted proteins, Ub-enriched proteins or DRibbles, respectively. One week after last vaccination, the lymphocytes collected from spleen and lymph node of vaccinated mice were added to 48-well plates (2.5 × 10^6^ cells/well) and re-stimulated for three days, in a 5:1 ratio, with EL4 or B16-F10 tumor cells pretreated with colchicine (1 μg/ml) for 2 hours. In addition, anti mouse CD3 antibody (BD Biosciences) was used as a positive control to induce the lymphocytes activation and IFN-γ secretion following the company protocol. Briefly, 48-well plates were coated overnight at 4°C with 10 μg/mL of anti-mouse CD3 prepared in sterile PBS. Then, the plates were washed three times with sterile PBS to remove non-bound soluble antibody and lymphocytes from the vaccinated mice were added. The level of IFN-γ in the cell culture supernatant was detected by ELISA. IFN-γ in the serum of the vaccinated mice was also measured by ELISA, whole blood were collected and kept for 30 minutes at room temperature to allow the clot formation. Then, different group’s sera were collected by refrigerated centrifuge (1300 rpm/4°C-Eppendorf AG 5417R) for 10 minutes.

To evaluate the antitumor efficacy of Ub-enriched proteins, C57BL/6 mice were s.c. injected with 1 × 10^6^ EL4 or B16-F10 tumor cells on day 0. Subsequently, Ub-enriched proteins, Ub-depleted proteins, whole cell lysates or PBS and Vx3(A7) protein (as control) were injected (i.n. s.c s.c.) into both flanks of tumor-bearing mice respectively on days 7, 9, and 11. Tumor growth was assessed by measuring the perpendicular diameters.

### Intracellular staining

Lymphocytes collected from spleen and lymph node of relevant vaccinated mice were cultured with inactivated B16-F10 tumor cells for 48 hours and in the presence of brefeldin A (10 μg/ml) for last 6 hours at 37°C in 5% CO_2_. Later, cells were washed in PBS supplemented with 3% fetal bovine serum (FBS) and blocked for nonspecific binding in 30% FBS for 30 min. Surface staining was performed using FITC-conjugated anti-CD4 and APC-conjugated anti-CD8 followed by intracellular staining with Cytofix/Cytoperm kit (eBioscience) in accordance with the manufacturer's instructions. Briefly, cells were fixed and permeabilized with Cytofix/Cytoperm solution for 1 hour on ice followed by washing in Perm/Wash solution. Next, cells were stained for 30 min on ice with PE-conjugated anti-IFN-γ. Finally, cells were resuspended in PBS buffer and analyzed by flow cytometry (FACSCalibur-BD Biosciences).

### Statistical analysis

Graphpad Prism 5.0 (Graphpad software, San Diego, CA) was used for all statistical analysis. The mean ± SD. was determined for each group in the individual experiments. The Student t-test was used to determine the significance of differences between vaccinated and control groups. *P*-values < 0.01 were significant.

## Results

### Ubiquitinated proteins are effectively isolated from tumor cell lysate by Vx3(A7) protein

**Figure 1:**
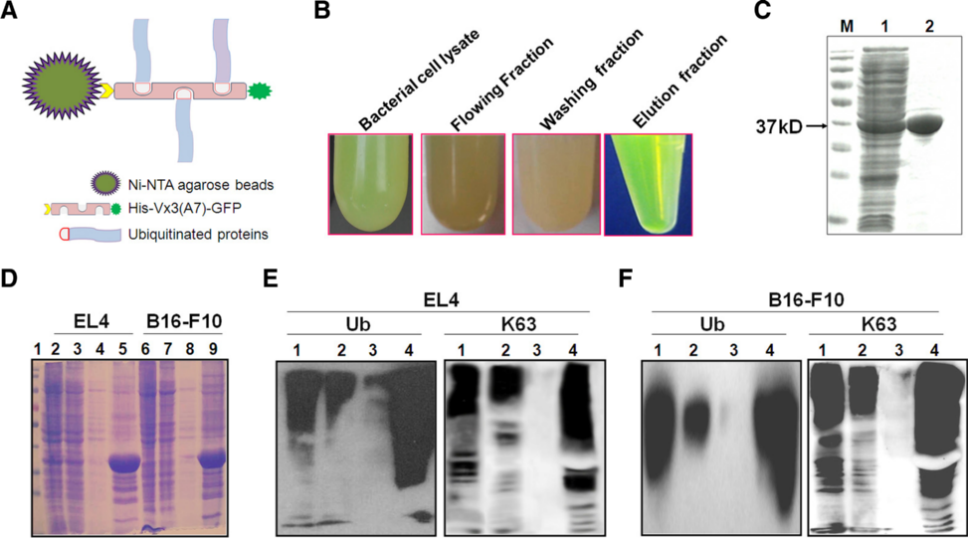
Ubiquitinated proteins were effectively isolated by Vx3(A7) protein. **(A)** A schematic diagram of Ubiquitinated proteins purification by binding with His-Vx3(A7)-eGFP protein conjugated with Ni-NTA agarose beads. **(B)** Ni-NTA agarose beads were used to purify Vx3(A7) protein, bacterial cell lysate, flowing fraction, washing fraction and elution fraction were collected and observed. **(C)** 12.5% SDS-PAGE was performed to detect the whole bacterial cell lysate proteins (lane 1) and the His-Vx3(A7)-eGFP fusion proteins (lane 2) which purified using Ni-NTA agarose Beads. **(D)** EL4 and B16-F10 tumor cells were treated with bortezomib (200 nM) and NH4Cl (10 mM). Whole cell lysates were prepared using tis-HCl lysis buffer. Ni-NTA agarose beads conjugated with Vx3(A7) proteins were used to isolate ubiquitinated proteins. Twenty micrograms of whole cell lysate (EL4, lane 2) and (B16-F10, lane 6), unbound (lane 3,7), washed (lane 4,8) and eluted fractions (lane 5,9) were loaded on SDS PAGE gel. **(E**, **F)** Western Blot analysis for ubiquitin proteins and K63 proteins in lysates (lane 1), unbound (lane 2), washed (lane 3) and eluted (lane 4) fractions harvested from EL4 **(E)** and B16-F10 **(F)** tumor cells using anti-ubiquitin and anti-K63 monoclonal antibodies, respectively.

We previously demonstrated that proteasome inhibition resulted in accumulation of ubiquitinated proteins, which were further shunted into autophagosomes after treatment with autophagy inducer, the following vaccination improved crosspresentation of the ubiquitinated proteins -containing autophagosomes [10,12,15]. Here, we used a ubiquitin binding protein Vx3 (A7) to isolate ubiquitinated proteins from tumor cells after blocking their proteasomal degradation pathway and sought to detect whether enriched ubiquitinated proteins could be directly used as a novel cancer vaccine (Figure 1A).

Vx3(A7) protein is composed from multiple tandem ubiquitin interacting motifs (tUIMs) with structured linker regions that bind with high-affinity to Lys63-poly ubiquitins [16]. Firstly, we used Ni-NTA agarose beads to purify his-Vx3(A7)-eGFP fusion protein expressed by E. coli DH5α that already transformed with pUbiG101-Vx3(A7)-eGFP recombinant plasmid. We found that the ‘Elution fraction’ was obviously greener than bacterial cell lysate, whereas the green color has not appeared in the ‘Flowing fraction’ and ‘Washing fraction’ (Figure 1B). These results were confirmed by SDS–PAGE analysis which showed that his-Vx3(A7)-eGFP fusion protein was successfully purified with an apparent molecular mass of 37 kDa (Figure 1C, lane M and 2) compared with bacterial cell lysate which revealed the presence of several proteins (Figure 1C, lane 1).

Next, we prepared lysates from EL4 and B16-F10 tumor cells in which proteasome function was inhibited by bortezomib, Ni-NTA agarose beads conjugated with the purified Vx3(A7) protein were used to isolate Ub-Ps. SDS-PAGE analysis revealed that the number of bands in the elution fractions were lower than those in the whole cell lysates and unbound fractions, whereas, few protein bands were detected in the washing fractions (Figure 1D). The western blot analysis using anti-ubiquitin antibody and anti-K63 antibody (Figure 1E and F) showed that the level of ubiquitin and K63 Ub protein in the unbound fractions (lane 2) and washed fractions (lane 3) was obviously lower than that in the eluted samples with same total protein concentration (lane 4). Moreover, much more ubiquitin proteins and K63 proteins were observed in the eluted samples from both EL4 and B16-F10 cells (lane 4) compared with whole cell lyaste (lane 1). These data indicated that Vx3(A7) protein is an effective tool to enrich ubiquitinated proteins (Ub-enriched proteins) from tumor cell lysate.

### Ub-enriched proteins induce tumor-specific immune response

**Figure 2:**
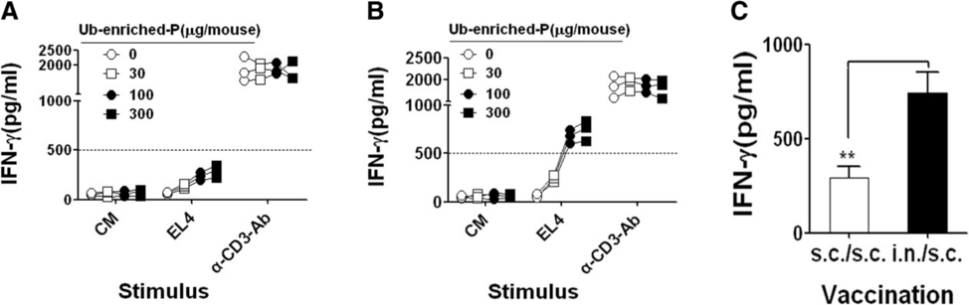
Ub-enriched proteins induced a tumor-specific immune response. C57/BL16 mice (3 mice/group) were vaccinated respectively with 0, 30, 100 and 300 μg by Ub-enriched proteins isolated from EL4 whole cell lysate via triple s.c. injections **(A)** or i.n. priming injection plus twice s.c. injections **(B)** with two days interval. On day 10 after last vaccinations, lymphocytes were collected from spleen and lymph nodes and re-stimulated by EL4 tumor cells that already inactivated by 10 nM of colchicine or without stimulation (CM). α-CD3-Ab stimulation was used as positive control. The IFN-γ produced by the responder cells was determined after 72 h by ELISA. **(C)** Comparing between the immune response resulted by previous two different route of vaccination (s.c. and i.n.) respectively using 100 μg of Ub-enriched proteins. Data are representative of three independent experiments results.

To investigate whether Ub-enriched proteins could induce tumor specific immune response, C57BL/6 mice were immunized with different doses (0, 30, 100 and 300 μg total protein / mouse- 3 mice/group) of Ub-enriched proteins collected from EL4 tumor cells via two different routes of administration, subcutaneous (s.c.) and intr-anodal (i.n.), respectively. On day 10 after last vaccination, the lymphocytes collected from spleen and lymph node of vaccinated mice were re-stimulated with inactivated EL4 tumor cells for 72 hours or cultured without stimulation (CM) (αCD3 mAb stimulation was used as a positive control), secreted IFN-γ was detected by ELISA analyses. As expected, vaccination with Ub-enriched proteins via both subcutaneous (s.c.) and intr-anodal (i.n.) injections induced specific immune response against EL4 tumor cells in a dose-dependent manner without a significant difference between 100 and 300 μg (Figure 2A and B). However, the lymphocytes from mice that primed with i.n. administration secreted a significantly higher level of IFN-γ than that from mice which primed with s.c. administration with the same dose of Ub-enriched proteins (Figure 2A-C). As shown in Figure 2C, the production of IFN-γ by lymphocytes from mice vaccinated s.c. with 100 μg of Ub-enriched proteins was markedly lower than that which vaccinated i.n with the same dose. These results indicated that Ub-enriched proteins were able to induce a tumor specific immune response in dose dependent manner. The priming via i.n. administration induced a stronger immune response than that via s.c. administration, Based on these findings, 100 μg Ub-enriched proteins via i.n. administration was used as a preferable strategy in the next experiments.

### Ub-enriched proteins elicit a stronger tumor specific immune response than whole tumor cell lysate and Ub-depleted proteins

In order to investigate whether Ub-enriched proteins could induce a stronger tumor specific immune response than whole tumor lysate or/and Ub-depleted proteins, we vaccinated four groups (3 mice/group) of C57BL/6 mice with Ub-enriched proteins, Ub-depleted proteins and tumor cell lysate prepared from B16-F10 and EL4 tumor cells, respectively. Injection with PBS was used as a negative control. The Blood serum and lymphocytes were collected from the vaccinated mice. Lymphocytes were re-stimulated for 72 hours with inactivated B16-F10 and EL4 tumor cells and with or without αCD3 mAb as a positive or negative control. The levels of IFN-γ in the serum and the culture supernatant were monitored by ELISA.

**Figure 3:**
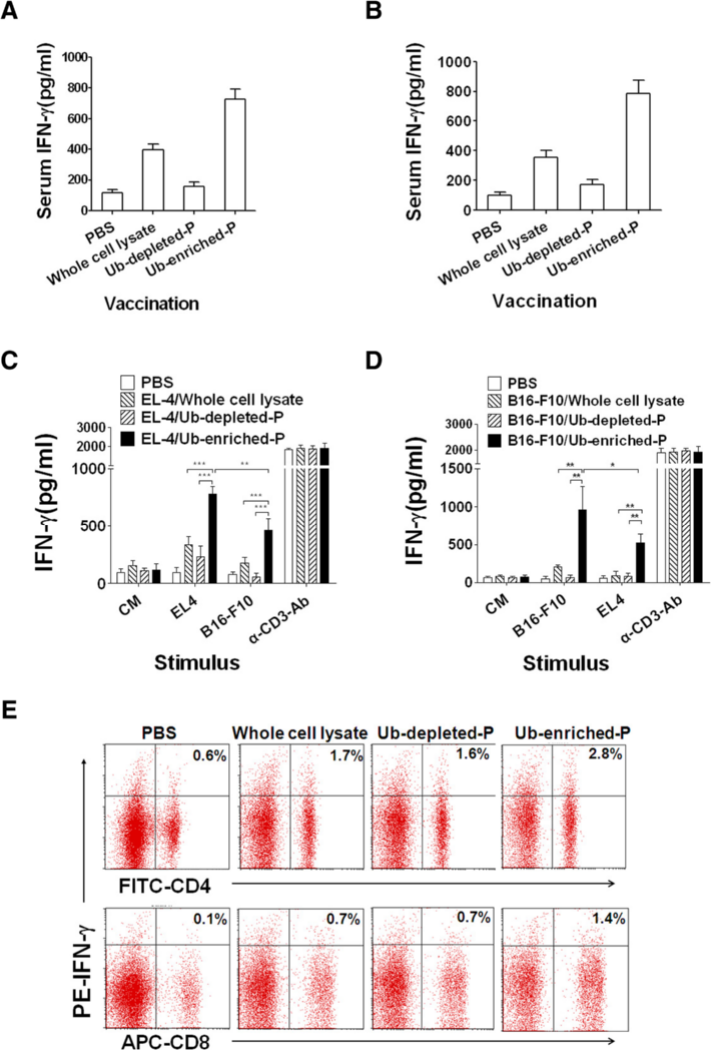
Tumor specific immune response resulted by Whole tumor cell lysate, Ub-depleted proteins and Ub-enriched proteins. C57/BL6 mice (3 mice/group) were primed by 30 μg i.n and twice 100 μg s.c vaccination with two days of interval by whole cell lysate, Ub-depleted proteins and Ub-enriched proteins from EL4 tumor cells **(A**, **C)** and B16-F10 tumor cells **(B**, **D)**. On day 10 after last vaccinations, blood was collected and the IFN-γ levels in the sera were detected by ELISA **(A**, **B)**. Lymphocytes were collected from spleen and lymph nodes and re-stimulated by EL4 and B16-F10 tumor cells that already inactivated by 10 nM of colchicine or without stimulation (CM). α-CD3-Ab stimulation was used as positive control. The IFN-γ produced by the responder cells was determined after 72 h by ELISA **(C**, **D)**. Data are representative of three independent experiments results. **(E)** Flow cytometric analysis of intracellular IFN-γ synthesized by CD4^+^ T cells (upper panel) and CD8^+^ T cells (lower panel) in the lymphocytes from mice vaccinated with PBS and whole cell lysate, Ub-depleted proteins and Ub-enriched proteins from B16-F10 tumor cells and restimulated with inactivated B16-F10tumor cells. Numbers within quadrants represent the percentage of positive cells for IFN-γ within the gate for relevant lymphocytes.

The results showed that IFN-γ level in the serum of mice vaccinated with Ub-enriched proteins from EL4 (Figure 3A) and B16-F10 tumor cells (Figure 3B) was higher than that in the serum of mice vaccinated with Ub-depleted proteins, whole tumor lysate or PBS. Furthermore, after stimulation with the relevant inactivated tumor cells in vitro, we found that lymphocytes from Ub-enriched proteins vaccinated mice produced a significantly higher level of IFN-γ than that from Ub-depleted proteins and whole tumor lysate vaccinated mice (Figure 3C, D). Interestingly, the lymphocytes from B16-F10-derived Ub-enriched proteins vaccinated mice produced a significantly higher level of IFN-γ than that from Ub-depleted proteins and whole tumor lysate vaccinated mice when stimulated with inactivated EL4 tumor cells (Figure 3C), similar results were observed when the lymphocytes from EL4-derived Ub-enriched proteins vaccinated mice were re-stimulated with inactivated B16-F10 tumor cells (Figure 3D). Intracellular staining detection showed that, after stimulation with B16-F10 tumor cells in vitro, the percentage of IFN-γ^+^ CD4^+^ T cells and IFN-γ^+^ CD8^+^ T cells from mice vaccinated with B16-F10-derived Ub-enriched proteins was higher than that from mice that vaccinated with tumor cell lysate, Ub-depleted proteins or PBS (Figure 3E). Together, these results indicated that Ub-enriched proteins vaccine is more efficient to induce a specific tumor immune response than Ub-depleted proteins and tumor cell lysate, supporting it as a tumor vaccine candidate.

### Both Ub-enriched proteins and DRibbles induce a strong tumor specific immune response

**Figure 4:**
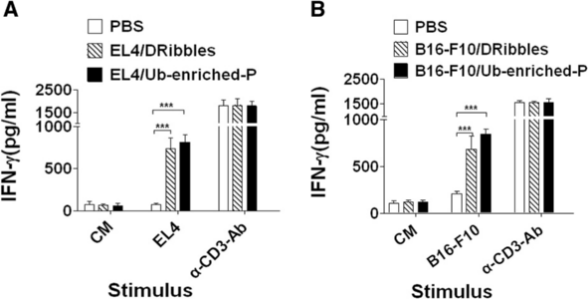
Tumor specific immune response induced by Ub-enriched proteins and DRibbles. C57/BL6 mice (3 mice/group) were primed by 30 μg i.n and twice 100 μg s.c vaccination with two days of interval by Ub-enriched proteins and Dribbles respectively isolate from EL4 cells **A)** and B16-F10 cells **B)**. Ten days after the last vaccination, Both lymph nodes and spleen of the mice were collected, processed into single cell suspensions and re-stimulated by EL4 and B16 tumor cells that already inactivated by 10 nM of colchicines or without stimulation (CM). α -CD3-Ab used as positive control. The secreted IFN-γ level was determined after 72 h by ELISA. Data are representative of three independent experiments results.

To prove the ability of Ub-enriched proteins to induce a tumor specific immune response same as DRibbles, three groups (3 mice/group) of C57BL/6 mice were vaccinated three times (i.n., s.c, s.c.) with Ub-enriched proteins and DRibbles from EL4, B16-F10 tumor cells and PBS as negative control respectively. Ten days after the last vaccination, lymphocytes were collected and stimulated with inactivated EL4 or B16-F10 tumor cells for 72 hours or without stimulation (CM). ELISA detection showed that there was no detectable difference in the level of IFN-γ secreted by the lymphocytes from DRibbles and Ub-enriched proteins vaccinated mice; both of them induced a production of a high level of IFN-γ compared with the negative control mice (Figure 4A, B). These data demonstrated that Ub-enriched proteins and DRibbles have a similar ability to induce a strong tumor specific immune response**.**

### Ub-enriched proteins has a potent therapeutic efficacy on established tumor model

**Figure 5:**
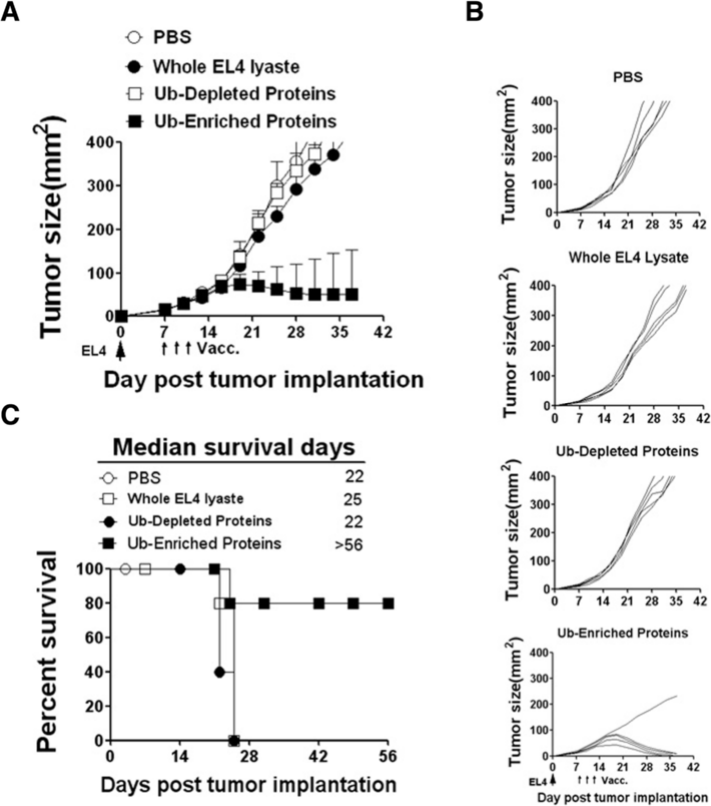
Anti-tumor efficacy of Ub-enriched proteins vaccine in EL4 tumor model. The anti-tumor efficacy of Ub-enriched protein vaccine was evaluated in EL4 murine models. **(A)** Twenty C57BL/6 mice were injected s.c. with 1 × 10^6^ EL4 tumor cells. Seven days later, mice established tumors were divided randomly into four groups of 5 animals each with comparable mean tumor sizes. One group was then left untreated; other three were received single i.n. and twice s.c. vaccination on days 7, 9 and 11 with EL4 cell lysate, Ub-depleted proteins and Ub-enriched proteins respectively. Tumor growth was measured three times a week. **(B)** Representing the tumor size of individual mice for each group. **(C)** The percent survival time of four EL4 tumor model groups. This data is from two experiments with similar results.

We evaluated the antitumor efficacy of the Ub-enriched proteins vaccinations in both EL4 and B16-F10 murine tumor models. In the EL4 model, twenty C57BL/6 mice were s.c. injected by 1 × 10^6^ of EL4 tumor cells. On day seven, tumor bearing mice were divided randomly into four groups and received single i.n. injection on day 7 and twice s.c. injections on day 9 and 11 with tumor cell lysate, Ub-depleted proteins, Ub-enriched proteins and PBS as a control. We observed that vaccination with Ub-enriched proteins significantly inhibited EL4 tumor growth compared with other three groups. Four out of five treated mice by Ub-enriched proteins rendered tumor free and survived more than 40 days (Figure 5A, B). We also found that Ub-enriched proteins prolonged the median survival time of the mice (more than 56 days for the Ub-enriched proteins group vs. 25 days for tumor cell lysate and 22 days for Ub-epleted proteins and PBS) (Figure 5C).

To further detect whether Ub-enriched proteins vaccination could not only inhibit the growth of homologous tumor, but also inhibit the nonhomologous tumor growth, we established two murine tumor models by s.c. injection of 1 × 10^6^ EL4 or B16-F10 into C57BL/6 mice (20 mice/established tumor model). On day seven, EL4 or B16-F10 tumor bearing mice were divided randomly into eight groups and received i.n. Priming vaccination on day 7 and twice s.c. vaccinations on day 9 and 11 with Ub-enriched proteins derived from EL4 cells and B16-F10 cells, respectively. Vaccination with PBS and Vx3(A7) protein was used as control.

**Figure 6:**
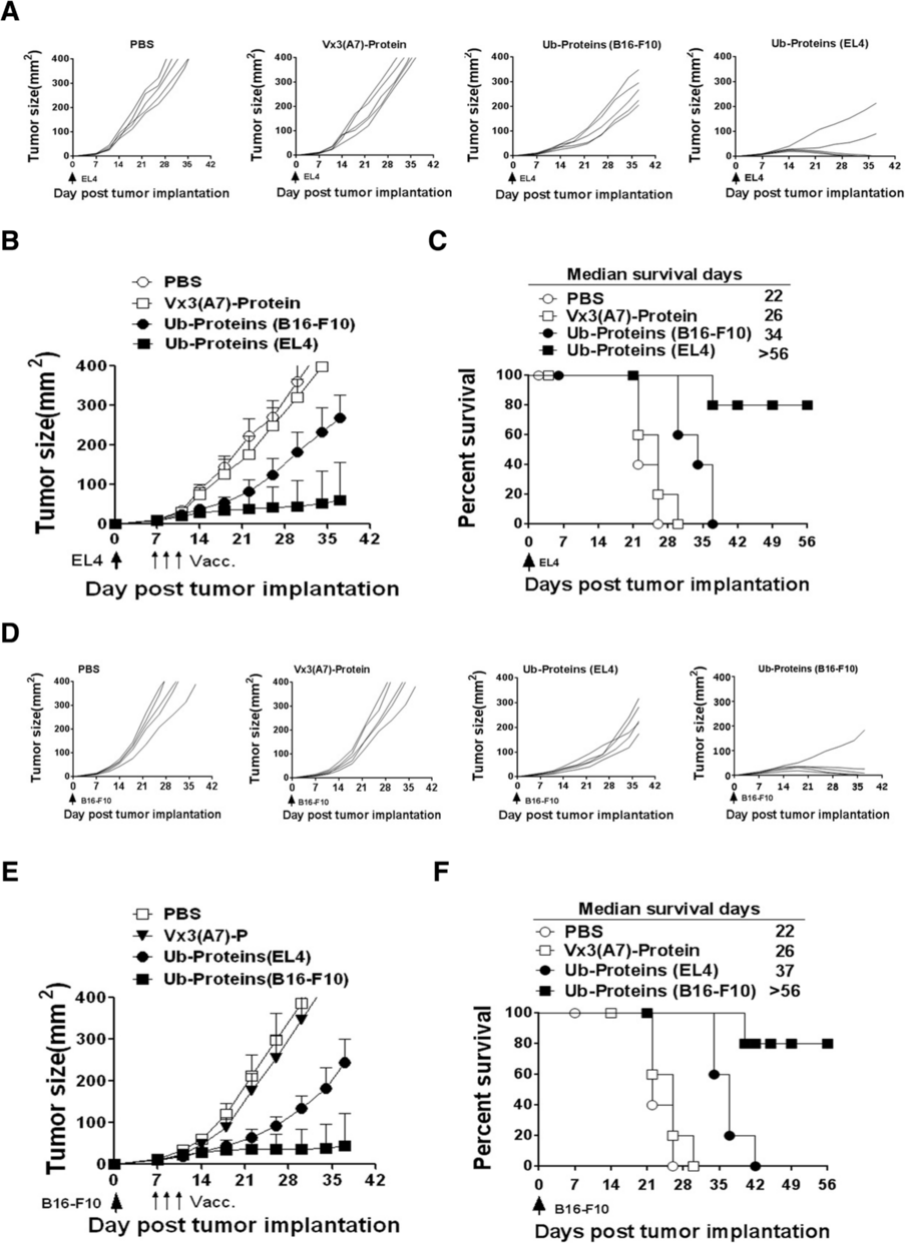
Anti-tumor efficacy and Cross Action of Ub-enriched proteins Vaccine in EL4 and B16-F10 tumor models. C57BL/6 mice were injected s.c. with 1 × 10^6^ EL4 tumor cells **(A**-**C)** and 1 × 10^6^ B16-F10 tumor cells **(D**-**F)**. Seven days later, mice established tumors were divided randomly into groups of 5 animals each with comparable mean tumor sizes. Two groups were then left untreated; other four were primed with single i.n. and twice s.c. vaccination n days 7, 9 and 11 with Vx3(A7) protein, Ub-depleted proteins and Ub-enriched proteins isolated from EL4 and B16 tumor cells respectively and separately. The tumor growth was measured three times a week **(A**, **B**, **D**, **E)**. **(A**, **D)** Representing the tumor size of individual mice for each group. **(C**, **F)** The percent survival time of EL4 and B16 tumor models groups respectively. This data is from two experiments with similar results.

As expected, EL4 tumor growth in 3 out of 5 treated mice with EL4-derived Ub-enriched proteins were regressed and rendered tumor free and survived more than 56 days compared with Vx3(A7) and untreated PBS controls. Interestingly, we found that vaccinations with B16-F10-derived Ub-enriched proteins significantly suppressed EL4 tumor growth and prolong the median survival time to 34 days compared with 26 days in the Vx3(A7) group and 22 untreated PBS control group (Figure 6A-C). Similar results were observed in the B16-F10 tumor model (Figure 6D-F), in which both vaccinations with B16-F10 and EL4 tumor cell-derived Ub-enriched proteins remarkably inhibited B16-F10 tumor growth and significantly improved survival and prolonged the median survival time to more than 56 days vs 42 days compared with 26 and 22 days in the Vx3(A7) and untreated PBS control groups respectively.

Moreover, 4 out of 5 treated mice with Ub-enriched proteins derived from B16-F10 cells were rendered tumor free. These results clearly demonstrated that Ub-Ps enriched from tumor cells have a potent anti-tumor efficacy on homologous tumor and expand a cross-protection on nonhomologous tumor.

## Discussion

Tumor antigen can be directly presented by tumor cells themselves or cross-presented by pAPCs. Both cross-presentation and direct presentation of tumor antigen have an important role in the induction of tumor-protective cytotoxic T lymphocytes immunity. Most of tumor cells, because of lacking costimulatory molecules, cannot induce naïve CD8^+^ T cells response by direct presentation and therefore cannot elicit protective antitumor immunity. In contrast, it has been documented that tumor antigen cross presentation by pAPCs is critical to induce antitumor immunity because pAPCs are able to deliver the antigen signal by MHC molecules and express a high level of co-stimulatory molecules signals simultaneously to the T cell for activation to occur [17-19].

Our previous studies have demonstrated that DRibbles isolated from tumor cells after proteasome inhibition, which lead to the accumulation of defective ribosomal products, short-lived proteins and their immunogenic fragments are efficient antigen carriers for cross-presentation by dendretic cells. DRibbles efficiently cross-prime antigen-specific naïve CD8^+^ and CD4^+^ T cells; and DRibbles vaccine induce an anti-tumor efficacy in different tumor models such as melanomas, lung carcinomas and breast carcinomas [11,13,20]. We found that the ubiquitinated TAAs are critical components of DRibbles which induce the antitumor efficacy. Moreover, it’s believed that much more ubiquitinated proteins could be collected from tumor cell lysates (especially after proteasome inhibition) comparing with DRibbles. So that, we hypothesized that the ubiquitinated proteins enriched from tumor cells after blocking their proteasomal degradation pathway could be an efficient tumor-specific antigen source for the cross-presentation. To confirm this determination, we prepared a ubiquitin binding protein Vx3(A7) in order to isolate the Ub-Ps from EL4 and B16-F10 tumor cells as antigen donor cells. The tumor specific immune response and antitumor efficacy resulted by the cross-presentation of Ub-Ps were assessed using EL4 and B16-F10 tumor models.

The proteasome is a large protein complex mediates the degradation of polyubiquitinated (abnormal) proteins [21]. Ubiquitin (Ub) is an 8.5-kDa polypeptide molecule tags misfolded or damaged intracellular proteins for degradation by ubiquitin–proteasome system [22,23]. The degradation of ubiquitinated short-lived proteins, including defective ribosomal products is catalyzed via the ubiquitin–proteasome system (UPS) espically in 26S proteasome sub-unit [9,24]. UBDs (ubiquitin-binding domains) are a group of modular proteins that bind non-covalently to Ub. Two main UBDs were identified, the UBA domain (ubiquitin-associated domain) and the UIM (ubiquitin-interacting motif). The UBA domain family is compact three-helical bundles. It is classified into four different groups according to ubiquitin-binding properties [25,26]. p62 (SQSTM1) is the well known example of UBA family. Its main action is recruiting Ub-Ps to the endosomes [27,28]. The UIM is a sequence motif which was first described in the 26S proteasome subunit PSD4/RPN-10 that is known to recognize ubiquitin [29,30]. S5a/Rpn10 is a subunit of the 26S proteasome and exists in the cytosol freely. It binds poly-Ub chains via its two Ub-UIMs [31]. Ubiquitin molecule has an ability to modify the target proteins by two different mechanisms, monoubiquitination or polyubiquitination [32,33]. Ubiquitin can be linked together through their surface lysine (K) residues (K6, K11, K27, K29, K33 and K63), forming poly-Ub chains of different lengths and shapes [34]. Few studies reported that Lys63-linked ubiquitin chains degradation is a proteasome dependent [35,36]. Whereas, many studies documented that Lys63- linked polyubiquitination has a destructive fate for its substrate through through a proteasome independent mechanism termed autophagy by which autophagasome is formed and fused with lysosomes to degrade the sequestered contents [37] In conclusion, Lys63-polyUb have non-proteasomal roles in DNA repair, autophagy and organelle clearance, endocytosi, and inflammation signaling [38,39].

Vx3(A7) protein is composed from multiple tandem Ubiquitin Interacting Motifs (tUIMs) with structured linker regions that bind with high-affinity to Lys63-polyUb. The main purpose of this protein is tracking the linkage-specific polyUb signals in yeast and mammalian cells and exploring their functions. Their specificity was determined both in vitro and in vivo [17]. Ub-Ps could be purified by different methods such as proteomic strategy combining of immunoprecipitation [40], or using a synthetic ubiquitin binding protein called tandem ubiquitin binding entities (TUBEs) [41]. In this study, we have confirmed that Vx3(A7) protein is an effective tool to enrich Ub-Ps from whole tumor cell lysate compared with other kinds of UBD proteins such as TUBEs (data not shown).

Our results showed that high concentration of Ub-Ps was purified from both EL4 and B16-F10 cell lysate using a native condition. This finding showed that Vx3(A7) protein is an excellent choice to enrich abundant level of Ub-Ps from cell lysate.

Our data refer to the ability of Ub-enriched proteins to induce a strong tumor specific immune response. More importantly, we found that the level of this response is increased dramatically by increasing the vaccine dose. Furthermore, our results clearly showed that the route of vaccination play a critical role in the inducing of tumor immune response, by which inguinal lymph nodes injection (i.n.) vaccination significantly induced a stronger immune response than subcutaneous vaccination.

Many studies reported that Inactivated tumor cells or tumor cell lysates are used as cancer immunotherapy [42,43], but it’s important to note that the majority of defective ribosomal products and short-lived proteins are not contained in inactivated whole tumor cells or tumor cell lysates vaccines due to rapid degradation. In this study we found that Ub-enriched proteins could induce higher immune response than whole tumor cell lysate and Ub-depleted proteins. Our findings also proved that Ub-enriched proteins and Dribbles have the same ability to induce a specific tumor immune response. Collectively the data points that Ub-Ps are important tumor specific antigens. One major advantage of our novel vaccine is the enrichment of higher concentration of ubiquitinated proteins from tumor cells lyaste than Dribbles. Moreover, isolation of ubiquitinated proteins from cell lysate is easier and faster than DRibbles.

Our previous results which demonstrated the anti-tumor efficacy of DRibbles prompted us to test our new therapeutic vaccine in murine lymphoma and melanoma tumor models. This study showed that vaccination with Ub-enriched proteins derived from EL4 or B16-F10 cells seven days after tumor establishment significantly inhibited EL4 and B16-F10 tumor growth respectively compared to whole cell lysate, Ub-depleted proteins and un-vaccinated group. Interestingly, dramatic regression and cure of established tumors were observed in 80% of the tumor bearing mice which vaccinated with Ub-enriched proteins. More importantly, our results showed that Ub-enriched proteins enriched from tumor cells not only have an anti-tumor efficacy in the homologous tumor but they also have the ability to delay the tumor growth and prolong the survival time of nonhomologous tumor. These results support a novel strategy for developing a tumor immunotherapy, and support further studies to test the efficacy of our vaccine in treating human tumors.

## Conclusion

These results support a novel strategy for developing a tumor immunotherapy depending on Ub-enriched proteins, and support further studies to test the efficacy of our vaccine in clinical trials.

## Abbreviations

Ub-Ps: Ubiquitinated proteins
DRibbles: DRiPs-containing blebs
pAPC: Professional antigen-presenting cell
TAAs: Tumor-associated antigens
t-UIM: Tandem ubiquitin interacting motif
EL4: C57BL/6 lymphoma cell line
B16-F10: C57BL/6 melanoma cell line
